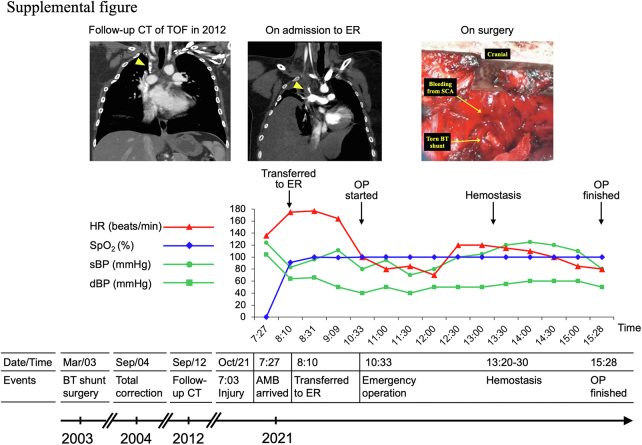# Traumatic Hemomediastinum and Hemothorax in a Patient With Totally Corrected Tetralogy of Fallot

**DOI:** 10.1016/j.atssr.2022.11.006

**Published:** 2022-11-18

**Authors:** Yuki Matsumura, Sho Inomata, Hikaru Yamaguchi, Masayuki Watanabe, Yuki Ozaki, Satoshi Muto, Naoyuki Okabe, Yutaka Shio, Yasuhiko Tsukada, Hiroyuki Suzuki

**Affiliations:** 1 Department of Chest Surgery, Fukushima Medical University, Fukushima, Japan; 2 Department of Emergency and Critical Care Medicine, Fukushima Medical University, Fukushima, Japan

## Abstract

An 18-year-old Japanese youth was transferred to our emergency department because of a traffic accident. He had a past history of total correction for tetralogy of Fallot as an infant. Chest computed tomography revealed hemomediastinum and hemothorax. In an emergency operation, massive bleeding from the mediastinum was observed. Bleeding arose from the torn Blalock-Taussig shunt and right subclavian artery. Hemostasis was achieved by clipping the shunt and suturing the subclavian artery. This is a rare case of a patient with hemomediastinum caused by torn Blalock-Taussig shunt after a high-impact accident who survived it by operation.

Tetralogy of Fallot (TOF) is a rare congenital heart defect characterized by 4 specific cardiac defects, namely, pulmonary stenosis, ventricular septal defect, right ventricular hypertrophy, and overriding aorta, that expands the aortic valve to allow blood from both ventricles to enter.^1^ If the patient has a decrease in oxygenation, surgical placement of a Blalock-Taussig (BT) shunt is first planned to secure passage of blood to the pulmonary artery.^2^ If the degree of cyanosis becomes relatively mild, radical operation is performed by approximately 1 year of age.^2^^,^^3^ Here, we describe a patient with totally corrected TOF in infancy who underwent emergency operation for traumatic hemomediastinum and hemothorax. Notably, the cause of bleeding was the BT shunt torn by a high-impact traffic accident, which led to massive bleeding from the pulmonary and subclavian arteries.

An 18-year-old Japanese youth was transferred to our emergency department because of high-impact trauma due to a traffic accident. He had a history of total correction of TOF in infancy and had been physically healthy when visiting our outpatient clinic 6 months before the injury (Figure 1). On arrival at the emergency department, he was conscious with relatively stable vital signs (Glasgow Coma Scale score, E3V5M6; heart rate, 136 beats/min; blood pressure, 124/104 mm Hg; oxygen saturation, unmeasurable because of coldness). His blood counts and biochemical test results did not show any severe abnormalities. Enhanced chest computed tomography (CT) revealed hemomediastinum and hemothorax (Figure 2). Although extravasation of contrast agent could not be detected, an emergency operation was performed because the vital signs were becoming unstable owing to massive bleeding.

**Figure 1:**
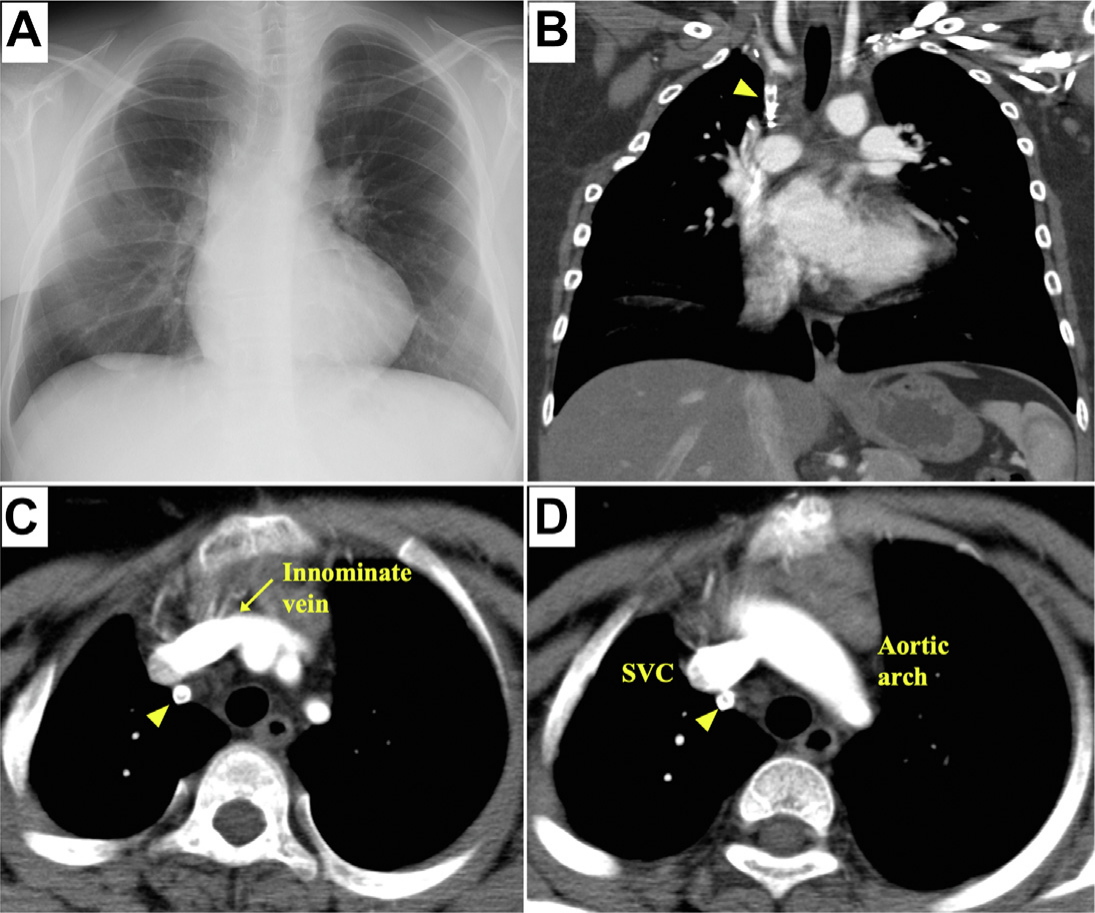
Radiologic images before the current trauma. (A) Chest radiograph 6 months before the injury had no apparent abnormality. (B-D) Chest computed tomography scans at 9 years old. (Arrowhead indicates Blalock-Taussig shunt; SVC, superior vena cava.)

**Figure 2:**
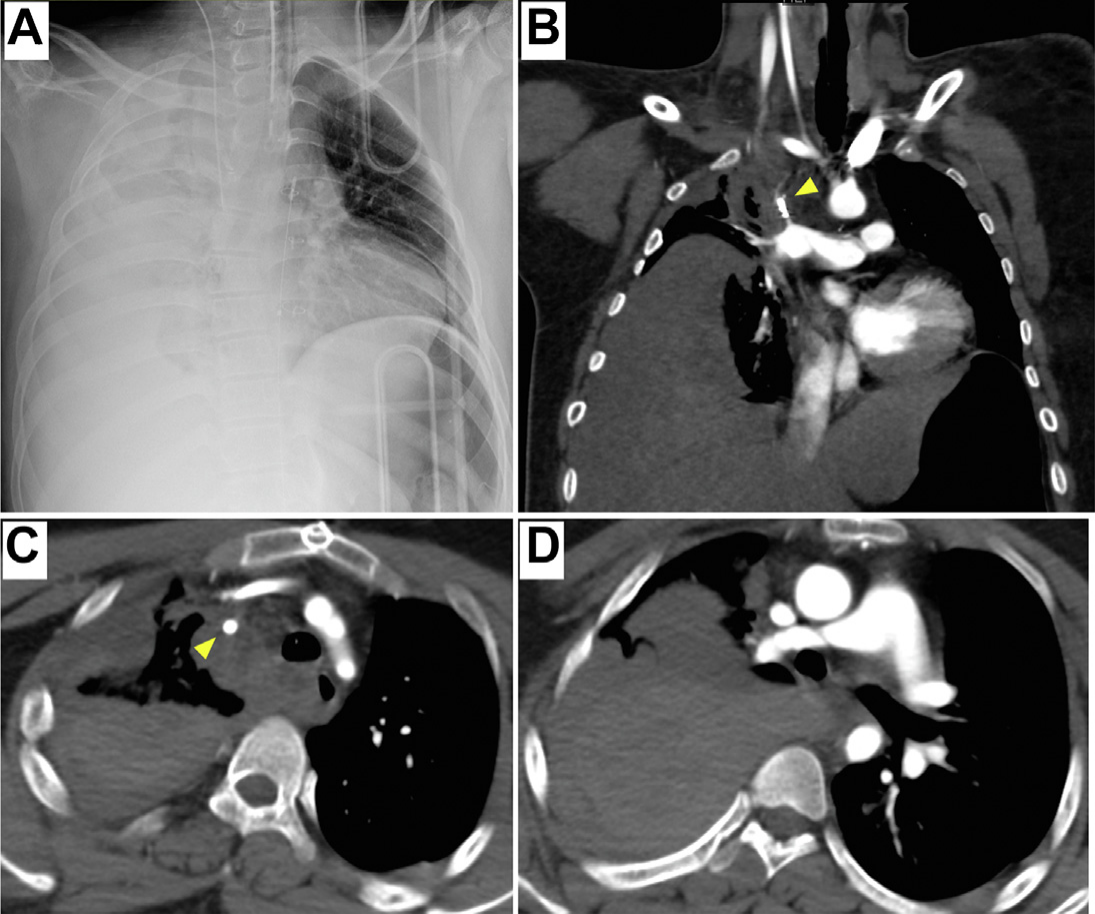
Radiologic images at admission. (A) Chest radiograph revealed no transparency in the right side of the chest, which implied hemothorax. (B-D) On chest computed tomography scans, subclavian artery side of Blalock-Taussig shunt (arrowhead) was disrupted, and fluid collection around it and right pleural effusion were observed.

Under right open thoracotomy, 500 mL of blood clot was pooled in the thoracic cavity, and retraction of the lung uncovered massive bleeding from the mediastinum onto which the right upper lobe was severely adherent (Figure 3; Supplemental Video). On dissection of the adhesion and compression of the mediastinum with the whole lung for hemostasis, bleeding from a tear in the BT shunt and right subclavian artery was discovered. After 20 minutes of compression achieved hemostasis, the subclavian artery was closed by suturing, and the torn BT shunt was clipped and covered with fractionated plasma products. Total blood loss was 10,020 mL, of which 3500 mL was returned by Cell Saver Elite (Haemonetics). Transfusion comprised 1680 mL of packed red blood cells and 2880 mL of fresh frozen plasma. During the operation, the patient’s vital signs and oxygenation were generally stable (Supplemental Figure). He was extubated on the day after operation, and postoperative hemorrhage was not observed. He was discharged on postoperative day 13 and has since been in good health when visiting our outpatient clinic.

**Figure 3:**
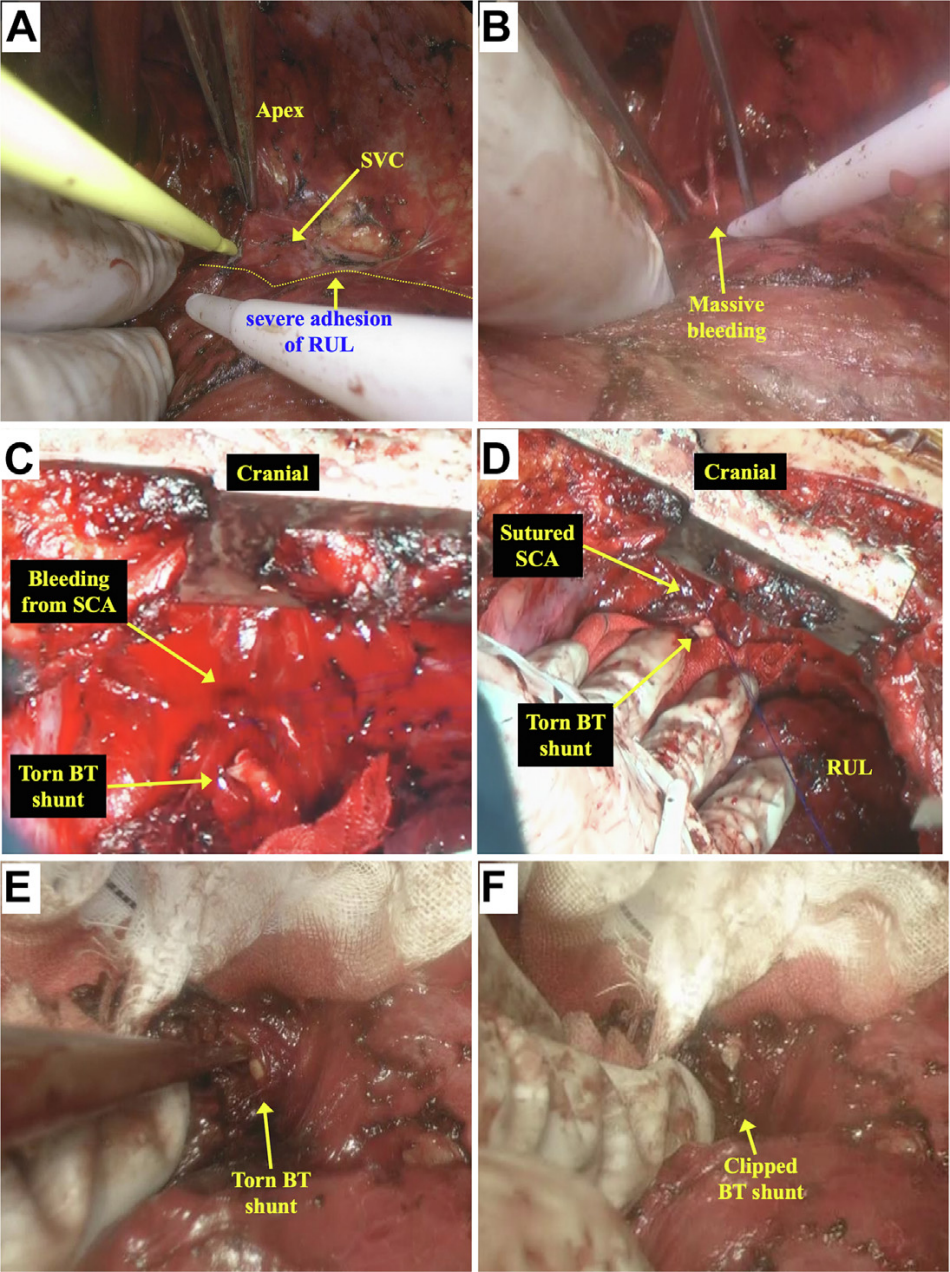
Intraoperative images. (A and B) Dissection of severe adhesion of right upper lobe (RUL) discovered massive bleeding from Blalock-Taussig (BT) shunt or right subclavian artery (SCA). (C and D) Bleeding from the torn BT shunt stopped by compression. Injury of the subclavian artery was repaired by suture. (E and F) The torn BT shunt was clipped with a surgical clip. (SVC, superior vena cava.)

## Comment

TOF is a rare congenital heart defect mainly characterized by pulmonary artery stenosis. If the patient has decreased oxygen saturation, BT shunt operation is initially performed to secure blood passage from the aorta to the pulmonary artery.^2^ Total correction is thereafter performed at approximately 1 year of age.^2^^,^^3^ This patient had undergone BT shunt operation as a newborn and total correction of TOF as an infant; 17 years later, the BT shunt was torn after a traffic accident, causing hemomediastinum.

Although there are many suggestive points for discussion about the patient’s clinical course, the most interesting question is why he was able to survive the interval until the operation (about 2 hours) despite that massive bleeding from the right subclavian artery and pulmonary artery arising from the torn BT shunt must have been present. Moreover, no extravasation of contrast agent was observed on chest CT scans. The bleeding origin was uncovered while dissecting adhesion of the right upper lobe, suggesting that this severe adhesion led to high internal pressure and closed the mediastinum, aiding hemostasis. Another question is why the BT shunt was torn into bleeding, even though the patient had no rib fractures or even apparent traumatic incised wounds except seat-belt marks. These facts indicate that the BT shunt was not damaged by simple external pressure, but the tear may have been caused by shearing force. Overtwist by the high-energy impact possibly overloaded the anastomosis of the BT shunt, which was then torn apart. During the operation, we discussed the indication of extracorporeal life support (ECLS) with cardiac surgeons, although ECLS is essentially contraindicated when uncontrollably massive bleeding is observed from great vessels.^4^^,^^5^ If ECLS had been applied when bleeding from great vessels still existed, it might have become more uncontrollable because of anticoagulation and high blood flow to the vessels.

We found no reports of bleeding from a BT shunt more than 10 years after total correction of TOF, with only 1 report of bleeding from the anastomosis just after construction of a BT shunt for TOF.^6^ Therefore, in this case, there was no expectation of torn BT shunt before the operation. In addition, we lacked knowledge of the details of the original operation for TOF, including even the existence of the BT shunt, which also made it more difficult to detect the origin of bleeding. Nevertheless, for future similar cases, reviewing the preoperative images provides us the following findings implying bleeding from a BT shunt. First, there is discontinuity of the BT shunt on CT scans, even though it can be tracked on previous follow-up CT. Second, hemomediastinum is observed around the edge of the torn BT shunt. Therefore, if we could have recognized the presence of the BT shunt and closely examined the CT scans, we might have predicted bleeding from the BT shunt before operation. In conclusion, we experienced an extremely rare case of traumatic hemomediastinum and hemothorax in a patient with totally corrected TOF 17 years previously. The cause of bleeding was the BT shunt torn by the high-impact accident, which led to massive bleeding from the pulmonary and subclavian arteries.

## Figures and Tables

**Supplemental Figure:**