# Supplementary material for: Traumatic Hemomediastinum and Hemothorax in a Patient With Totally Corrected Tetralogy of Fallot
Source: Ann Thorac Surg Short Rep. 2022 Nov 18;1(1):91–3. doi: 10.1016/j.atssr.2022.11.006 (PMC11708281; doi:10.1016/j.atssr.2022.11.006)
Supplement: Supplemental Figure Legend [file mmc2.docx]

**Supplemental Figure 1.** Timeline of the patient. The patient’s vital signs were generally

stable during the timeline. AMB, ambulance.
